# Supplementary material for: Are carbon emissions trading and green financial instruments synergistic? -Comprehensive quantitative research based on content analysis
Source: PLoS One. 2024 Mar 7;19(3):e0298601. doi: 10.1371/journal.pone.0298601 (PMC10919601; doi:10.1371/journal.pone.0298601)
Supplement: S1 Data — (ZIP) [file pone.0298601.s001.zip › Data/地级市Z_F工作报告.zip!/╔╜╬≈/╜·╓╨╩╨/└┤╫╘╣1⁄2╓┌║┼í╛┬φ┐╦╩2╛▌═°í┐_2013.docx]

各位代表：

　　现在，我代表市人民政府向大会报告政府工作，请予审议，并请市政协委员和其他列席人员提出意见。

一、2012年工作回顾

　　2012年，是我市经济社会发展取得重要成果的一年。我们坚持以建设全省“四化”率先发展区为统领，抓住全省综改试验区建设和太原晋中同城化两大机遇，围绕项目推进、同城发展、综改试验、民生改善、民主法治等工作重点，推动转型跨越，有效应对复杂多变的宏观环境和经济下行的严峻形势，实现了经济平稳较快发展，全市地区生产总值完成985.9亿元，增长10.2%；规模以上工业增加值480.9亿元，增长13.7%；财政总收入210.4亿元，增长18.1%，其中一般预算收入99.2亿元，增长27.2%。投资、消费、出口拉动有力，固定资产投资744.1亿元，增长28.2%；社会消费品零售总额377.3亿元，增长16%；外贸进出口总额4.9亿美元，增长112.2%。居民收入持续增长，城镇居民人均可支配收入21409元，增长13.2%；农村居民人均纯收入7936元，增长14.8%。9项主要指标中，社会消费品零售总额增幅与全省持平，其余8项增幅均高于全省平均水平。市三届人大三次会议确定的地区生产总值、工业增加值、社会消费品零售总额，因受宏观经济形势下行压力加大的影响，没有完成，其它预期指标和约束性指标都已完成，市政府向全市人民承诺的实事全部兑现。

　　一年来，市政府主要做了以下工作：

　　一是着力推进项目建设，发展后劲更加坚实。深入开展“项目落地年”活动，围绕建立机制、创优环境、要素保障等关键环节，推动招商引资，推进联审联办，加强督查考核，项目推进取得新进展。全市签约项目总投资2399亿元，项目落地1432.8亿元，均超额完成市定任务；市级重点建设项目年度投资完成额、投资计划完成率好于上年；共有134个重点项目完工或基本完工，太重榆液高端液压产业园区、平遥煤化宇皓新型光学材料产业园、《又见平遥》等具有全局意义的高端前沿项目建成投产，对全市转型跨越发展的支撑和引领作用逐步凸现。传统产业改造实现新突破，煤矿升级改造完成投资100亿元，新增8个生产矿井竣工，新增21个联合试生产，原煤产量突破8000万吨；焦化企业兼并重组全面启动，推动产能向省定兼并主体企业集中。新兴产业培育取得新进展，轻型直升机、新能源汽车等制造项目扎实推进；新材料产业健康发展，光学材料开始试生产；太铁货运中心前期工作进展顺利，两个中央厨房投产运营；文化产业增加值占GDP比重达到5.36%，继续保持全省领先；旅游总收入216亿元，增长59.7%。特色现代农业建设迈出新步伐，增产粮食1.1亿公斤，新发展特色专业村269个、设施蔬菜6.2万亩、各类养殖小区（园区）237个、干果经济林12.1万亩；农产品加工龙头企业达到345户，实现销售收入92.6亿元，增长21.2%，转化农产品43亿公斤，带动51万农户增收8.1亿元；农民专业合作社向标准化发展，组织管理、服务功能、带动作用得到进一步加强；左权绵核桃、太谷壶瓶枣等5件商标被国家工商总局注册为地理标志证明商标，全市达到9件，居全省第一。园区承载能力有了新提升，榆次工业、介休循环经济生态工业等园区产业进一步集聚，实现销售收入占全市工业的43%，同比提高6个百分点；晋中经济开发区升级为国家级经济技术开发区。

　　二是着力统筹城乡发展，城乡面貌明显改观。我市城镇化工作全省领先，城乡基础设施投入创历史新高，城镇化率达到47.41 %，提高1.67个百分点。农村街巷硬化、便民连锁商店、文化体育场所和中等职业教育免费、新型农村社会养老保险新“五个全覆盖”在全省率先完成。太原晋中同城化取得新进展，市城区市政重点工程完成投资141.2亿元，高出上年1倍多，山西高校新校区拔地而起，基础设施和配套服务建设加快推进，新生开始入住；新建四条纵向城市道路与太原延伸对接，“东出口”工程、晋商公园二期、潇河湿地公园一期等项目竣工，新开902、903两路同城公共交通。重大基础设施建设步伐加快， 以东山供水、中部引黄、松塔水电站、恋思和石膏山水库、松溪河供水工程建设为标志，水利建设“百项工程、百亿投入”进展顺利；龙城高速、汾邢高速平遥左权段、省道汾屯线晋中段竣工通车，董榆线和顺段一级公路改建开工；电网及农网改造工程新增35千伏及以上线路117.5公里，新增变电容量736兆伏安。节能减排和生态建设扎实推进，全市单位地区生产总值能耗同比下降3.53%，二氧化硫、化学需氧量、氨氮、烟尘、粉尘排放量分别削减1.35 %、1.37%、1.1%、6.47 %、7.19%，氮氧化物增排1.66%；市城区空气质量优良率98.8%，居全省第一，各县（区、市）空气质量优良率均在97%以上；新造林41.2万亩，林木绿化率提高1.1个百分点，介休市在全市首家跨入国家园林城市行列，汾河湿地森林公园被确定为国家级湿地公园试点，昔阳县被命名为省级园林县城，灵石县通过国家卫生城市综合评审，左权县被省政府命名为林业生态县，全市11个县（区、市）继续保持了省级卫生城市称号。

　　三是着力改善民生民利，社会事业全面发展。全市财政用于民生领域支出较上年增长13.18%。保障性住房开工建设44537套，竣工16057套，居全省第一，2498户农村危房改造工程全部竣工。住房公积金支持保障性住房全国试点城市申报成功。省级创业型城市创建活动扎实开展，城镇新增就业4.48万人，城镇登记失业率1.94%。各类保险参保人数、保费征缴额进一步提高，城乡低保实现应保尽保。全市80周岁以上的城乡居民免费享受基本医疗保险。“爱心煤”政策得到落实，86.42万户农民家庭受益。义务教育均衡发展和学校标准化建设继续走在全国前列，教育信息化工作成为全国首批试点市，教育公平步伐坚实。公立医院改革试点工作扎实，基本药物制度在乡村卫生院（室）全面实施；新农合参合率99.14%，9个县（市）推行了城乡困难群众医疗救助“一站式”服务。县级体育馆全覆盖全面启动，城市社区500米健身圈工程70个社区、160块健身场地交付使用。文化体制改革、文艺创作以及文物和非物质文化遗产保护传承取得新进步。持续加大扶贫开发力度，贫困人口减少2.82万人，农民增收明白卡覆盖全市60%行政村和70%农户。安全生产风险防控试点启动，“打非治违”专项行动有力推进，安全生产事故起数比上年下降17.7%，死亡人数下降8.06%。有力推进食品链条全过程风险预防控制，继续坚持全过程监督问责，责任监督促进食品安全得到保障。深入开展国防教育和双拥工作，积极发展人口计生、妇女儿童、残疾人、老龄事业和红十字会、慈善等公益事业，民族、宗教、外事、侨务工作进一步加强，气象、地震、人防和档案、史志、科普等工作都取得新成绩。

　　四是着力推动改革创新，综改试验稳步实施。认真组织20个重大转型标杆项目，投资达到140.8亿元，完成年计划130.7%。全省资本市场改革创新示范区建设取得新进展，5家村镇银行挂牌营业，3家改制农村商业银行健康运营，3家股权投资管理公司挂牌成立，2支私募股权投资基金设立我市，新增2户企业在天津股交所挂牌，全市在资本市场直接融资60亿元，民营资本进入金融领域56.5亿元。建设用地渠道增加，11个县（区、市）全部列为城乡建设用地增减挂钩试点，获得周转指标6000亩，新造耕地、复垦土地、盘活存量土地都取得较大进展。市级投资建设项目联合审批改革成效明显，76个市级投资项目审批时限由100个工作日压缩为43个工作日。扩权强县试点工作稳步推进，省定市属经济社会管理权限全部落实到县。

　　五是着力促进社会和谐，民主法制和精神文明得到加强。市政府自觉接受市人大及其常委会的法律监督和市政协的民主监督，积极支持各民主党派、工商联、无党派人士参政议政。办理人大代表意见建议232件、政协委员提案319件，办结率100%。加快推进“法治晋中”建设，全面实施“六五”普法；加强法治政府建设，认真落实廉政建设责任制，积极推进政务公开，政务环境不断优化，政府公信力、执行力进一步提升。深入开展群众性精神文明创建活动，完善安全监管、社会管理和居民自治网格化，加强知责、履责、问责教育，着力排查化解各类社会矛盾，依法打击违法犯罪活动，社会保持和谐稳定，加强和创新社会管理工作经验在全省、全国推广。

　　各位代表,过去的一年，我市继续保持了经济社会平稳较快发展势头，实属来之不易。这是中共晋中市委统揽全局、正确领导的结果，是市人大、市政协大力支持、有效监督的结果，是全市人民辛勤工作、共同努力的结果。我代表市人民政府，向全市人民，向驻地部队广大指战员、武警官兵、公安干警和国家、省驻地单位，向各民主党派、工商联、各人民团体和社会各界人士，向所有关心和支持晋中发展的同志们、朋友们，表示诚挚的感谢！

　　我们清醒地认识到，我市经济社会发展中还存在不少矛盾和问题。主要是：经济总量不大、结构不优、质量效益不高，传统产业层次较低，新兴产业比重偏小，中小企业发展不足；重点项目数量少体量小，招商引资力度不够；节能减排和环境保护任务艰巨，自主创新能力不强，发展方式还比较粗放；安全生产基础不牢，责任落实仍有盲点，实现全市安全生产形势稳定好转任重道远；城乡区域不平衡，社会事业欠账较多，农民增收特别是贫困地区脱贫致富任务艰巨；发展环境有待进一步优化，行政不作为、慢作为、乱作为现象时有发生；政府职能转变滞后，思想解放不够，形式主义、官僚主义乃至少数干部失职渎职、以权谋私问题依然存在。我们必须采取更加有力的措施认真加以解决。

　　二、2013年主要工作

　　2013年是全面贯彻落实党的十八大精神的开局之年，是实施“十二五”规划承前启后的关键一年，是为全面建成小康社会奠定坚实基础的重要一年，也是全面推进转型综改试验区建设的重要一年。做好今年工作意义重大。为此，要全面贯彻落实党的十八大精神，以邓小平理论、“三个代表”重要思想、科学发展观为指导，围绕建设全省“四化”率先发展区总目标，按照“速度快、效果好、特色浓、人民富”总要求，紧紧抓住转型综改试验区建设和太原晋中同城化两大机遇，坚持开放引领、项目支撑，加快推进工业新型化、农业现代化、市域城镇化、城乡生态化和文化旅游大市建设，全面加强经济建设、政治建设、文化建设、社会建设、生态文明建设，团结带领全市人民，解放思想，改革开放，凝聚力量，攻坚克难，建设转型发展先行区、晋商文化复兴地、现代宜居生态市，为全面建成小康社会努力奋斗。

　　主要预期指标是：地区生产总值增长10%以上，规模以上工业增加值增长12%以上，固定资产投资增长25%，社会消费品零售总额增长15%，外贸进出口总额增长6%，财政总收入和一般预算收入分别增长18%和12%，城镇居民人均可支配收入增长10%，农村居民人均纯收入增长14%，城镇新增就业3.87万人，城镇登记失业率控制在4%以内，居民消费价格涨幅控制在3.5%左右。

　　约束性指标是：万元GDP综合能耗下降3.5%，万元GDP二氧化碳排放量下降3.7%，二氧化硫、化学需氧量、氨氮、氮氧化物排放量分别下降2.5%、2.7%、3.5%、10%，烟尘削减0.5%，粉尘削减0.3%，万元工业增加值用水量下降5.2%。

　　实现以上指标，要重点抓好八个方面的工作：

　　（一）不遗余力推进项目建设，努力增强发展后劲。

　　坚持把项目建设作为转型跨越发展的第一要务，全面推进项目储备、签约、落地、开工、建设和投产，确保今年全社会固定资产投资完成930亿元。扩大招商引资。立足资源禀赋、区位优势和产业结构，充分发挥企业招商引资主体作用，充分发挥开发区、各类园区招商引资排头兵作用，深化以优势资源换项目、换投资、引技术、引人才，强化与先进发达地区的交流合作，拓展与央企、国企和大型民企的合作领域，积极争取国家部委和省直部门更多的项目支持，将政策能量转化为实体项目，力争引进建设更多的转型大项目，力争更多好项目进入中央和省投资计划。积极参加“中博会”、“厦洽会”、“广交会”等重大招商活动。全年招商引资1800亿元以上。

　　强化服务保障。由市县两级审批、核准和备案的项目，联动服务要进一步优化流程、减少环节、缩短时间。要在严格保护耕地的同时，精心组织推进用地改革，加快土地整理收储，盘活闲置存量，确保土地供应满足项目推进需要。要积极帮助项目筹措资金，充分发挥政府、企业、金融机构和社会多方面的积极性，推动金融服务创新，确保资金落实到位。

　　完善推进机制。进一步落实领导干部联系重点工程制度，明确任务、时限和标准，责任到人。坚持每月调度制度，及时协调解决突出困难和问题，公布工程进度。创新执行直通车制度，对中央和省属企业投资我市的项目和总投资10亿元以上的重点工程，遇到重大问题和事项，属地县（区、市）政府必须在24小时内直接报送市政府责任人手中，必须在接手后24小时内提出处理意见、予以答复。严格目标责任考核制度，层层细化分解项目任务，明确责任主体和时限要求，强化考核，严格兑现。

　　（二）抢抓机遇加快同城发展，努力提升城镇化质量。

　　坚持以城镇化为引领，高举太原晋中同城化龙头，加快城乡一体化建设，努力取得新突破。加大城镇建设力度。全面推进市城区扩容提质，重点实施九大类89项市政工程项目，今年完成120亿元以上投资。完善路网建设，打通乌金路,延伸文华街、大学街，拓展迎宾街、安宁街等7条道路；完善市政设施，重点组织引黄管网建设等8项城市供水工程，完成污水处理厂二期工程和市城区生活垃圾卫生填埋处理厂完善工程；以医疗、商业网点等为重点，加快高校新校区配套服务设施建设；加快建设大型公共和标志性建筑，力争完成规划展示馆主体工程，全面启动博物馆、图书馆、民俗风情街、汇通大厦建设；实施片区改造工程，重点改造羊毫街片区和聂村、龙田、北关、大东关等城中村，整体搬迁小南庄。深入实施“大县城”战略，按照“一区两翼”总体布局，加快城镇集群发展，重点推进7个县城新区和12个省级示范镇基础设施建设，全市完成50条城市小街巷维修改造和30条城市小街巷亮化任务。

　　加大城乡基础设施建设力度。协调保障汾邢高速左权至和顺省界段、阳黎高速阳泉至左权段年内通车运营，左权至黎城段加快建设，力争今年最后两个县通高速，争取上马319省道左权县城过境段改线工程；实施农村公路改造150公里，新增公路通车里程200公里，建成晋中汽车客运总站。配合做好东山调水、中部引黄工程协调工作，规划建设东山调水、中部引黄受水县（市）区域小水网，创造同期供水条件；完成松塔水电站、恋思和石膏山水库附属设施工程，加快推进松溪供水工程建设；完成11座小型水库除险加固，争取3座中型水库除险加固项目；推进5个中央财政小型水利重点县建设。新开工灵石两渡220千伏、榆次新付110千伏等6个电网建设项目，完成榆次北500千伏和介休东湖龙220千伏等12个续建项目，实现新增变电容量335.9万千伏安；继续推进新一轮农网改造升级，完成6个35千伏增容改造项目、26个10千伏及以下中低压项目。

　　加大城乡一体化公共服务力度。落实放宽城市户口条件的相关规定，执行好按实际居住地登记户口的政策，推行居住证制度，解决好社会保障、子女上学、保障性住房等问题，有序推进农业转移人口市民化，使农民进得来、留得住、发展好。加快数字城管建设，推动城市管理全面融入社会管理网格化，着力构建市、县、乡（镇）、村（社区）网格管理责任体系，加快公共安全视频监控系统建设步伐，着力城市精细化管理，有效解决交通拥堵、环境脏乱等突出问题。

　　（三）坚持不懈深化结构调整，努力加快产业转型步伐。

　　坚持以煤为基、多元发展，加快构建具有晋中特色的现代产业体系，促进经济转型发展。改造提升传统产业。坚持技术改造和兼并重组两手抓，加快实施煤矿技改项目，推进矿井机械化、信息化、标准化、智能化步伐，力争原煤增产1000万吨。以化产引领推动焦化行业兼并重组，抓好主体企业新上大机焦及配套化产深加工项目前期工作，理顺重组主体企业产权关系，重点抓好介休、灵石两个千万吨级焦化集聚区建设。坚持集群规模发展方向，以建设院士工作站、园区公共服务平台、筹建国家液压产品质检中心为重点，进一步加快榆次液压产业园建设步伐；着力开发高档新型纺机产品，年内建成国家纺机质检中心；围绕打造国家外贸转型升级示范基地、国家玻璃产品质检中心、中华玻璃器皿博物馆三大“国”字号工程，继续完善大华宏艺、下申、温曲三个玻璃器皿基地建设。以提升铸造产业自动化水平和产品档次为方向，重点实施峰岩15万吨汽车配件铸件、恒达精密铸造等项目，加快建设太谷玛钢铸造园区。

　　培育壮大新兴产业。坚持产品研发和市场开拓两手抓，加快发展装备制造业，加快推进山西新能源汽车、中航美运特种车、介休青云轻型直升机等制造项目，集聚零配件制造产业，推动建立汽车物流贸易中心。大力发展新材料产业，加快东方希望铝系产品、平遥宇皓纸业改性材料、昔阳铁氧体永磁材料、寿阳新型环保石头纸等项目建设，力争太钢万邦铬铁等项目竣工投产。以平遥宇皓2万吨导光板投产项目为基础，上下游延伸产业链。

　　加速发展现代服务业。坚持放大优势和重点突破两手抓，深化文化体制改革，大力实施文化惠民工程，加快市级“五馆一院”和县级“三馆一院”建设步伐。强化文化产业发展的政策支持和项目支撑，抓好国家级晋中文化生态保护实验区晋中区域相关工作，着手规划市城区文化产业创意园。推进文化旅游深度融合、互动发展，精心营销《又见平遥》，启动平遥古城创建5A景区，实质性推进乔家大院、绵山创建5A景区，推动麻田八路军总部纪念馆提档升级；大力推广旅游标准化，引深示范城市创建和平遥县示范县建设；完善要素体系，整治卫生环境，整顿市场秩序，提升管理水平。加快太铁货运中心、方略保税晋中延伸工程等项目的各项前期工作。精心组织全国家政服务体系建设试点工作，完善万村千乡市场工程，升级改造5个县级商品配送中心，新建改造6个乡镇区域商贸中心；推进放心餐工程，力争放心餐点覆盖主城区60%以上；申报启动国家肉类蔬菜流通追溯体系建设项目。

　　增强科技创新驱动发展能力。加快构建以企业为主体、市场为导向、产学研用相结合的技术创新体系，完成“太榆科技创新城”晋中规划，争取一批省级研究设计院所项目早日落地。依托榆次工业园建设晋中科技创新园，年内完成中心区基础设施建设，筹建市级创新创业服务中心。依托晋中经济技术开发区启动海归创业中心。发挥企业技术创新主体作用，扶持龙头企业技术攻关，力促方盛液压院士工作站建成运行，依托金谷铸造、鸿基科技、红海玻璃、斯普瑞机械制造等核心企业，年内完成四个行业公共技术研发中心建设。

　　（四）毫不放松抓好“三农”工作，努力增加农民收入。

　　坚持把“三农”作为全部工作的重中之重，认真落实中央一号文件精神，千方百计让农业强起来、农村美起来、农民富起来。大力发展特色现代农业。坚持“一村一品”主攻方向，围绕“八个一”建设标准，落实11个精品片区规划，巩固提升存量542个专业村和26个专业乡镇，新发展200个达标专业村、10个专业乡镇，新发展小杂粮示范基地10万亩、干鲜果经济林19万亩、设施蔬菜和苗木各6万亩，新建规模养殖小区200个。以昔阳双孢菇和榆社云安村两个国家级农业标准化示范项目建设为突破口，加快东山规模园区建设步伐。深化晋中现代农业示范区9个省级项目，重点抓好榆次南湖、祁县西六支设施蔬菜2个标杆项目，新完成投资10亿元。强化与中国农大、山西农大、山西农科院等院校合作，建立果树、蔬菜、养殖、加工等10个科研示范中心，加强基层农技服务体系建设，搞好“十百千万农民大培训工程”。推动“513”龙头企业加快发展，重点扶持晋中金粮农科、祁县千朝现代农业示范园等大项目建设，支持平遥龙海、冠云牛肉、太谷巨鑫伟业农业示范园等与国内知名大企业、大集团对接，有效整合联接生产、加工、销售各个环节，新增一批“龙头企业+基地+中介组织+农户+标准化”组织形态，全市农产品加工流通销售收入突破110亿元。大力支持发展多种形式的新型农民合作组织，规范建设650个省、市、县三级农民专业合作示范社，培育专业大户、庄园经济、家庭农场等规模经营主体100个。加大农民增收明白卡推广力度。实施4000个农户科学储粮专项行动。

　　扎实推进新农村建设。坚持产业集聚、设施共享、人口集中、功能完善的基本要求，启动10个新农村集中连片示范区和100个“六美”农村建设，全面完成270个重点新农村“四化四改”和1438个行政村街道亮化任务。捆绑使用土地增减挂钩等各类支农资金，加快旧村改造步伐，推进新型农村社区建设。

　　进一步抓好扶贫开发。今年要全力确保3.2万以上贫困人口脱贫，完成易地扶贫搬迁3760人。加快实施昔阳、榆社两县6万亩核桃经济林开发项目，完成左权核桃、和顺肉牛片区扶贫开发项目验收任务。抓好左权、和顺20个村整村推进项目。资助贫困地区274名困难高中、大学生完成学业。提升16个国家、省扶贫龙头企业带动作用。培训贫困地区农村劳动力3360人，就业转移率达到70%以上。继续推进非贫困地区11.34万贫困人口“两项制度”有效衔接和后续扶持工作。深化干部包村增收和机关定点扶贫活动，扎实推进城乡“双联共建”活动。

　　（五）下大力气推进生态文明建设，努力打造美丽晋中。

　　坚持节约保护优先、自然恢复为主，推进绿色、循环、低碳发展，让晋中天更蓝、地更绿、水更清。狠抓节能降耗。开展“双百户”重点能耗企业节能行动，认真落实节能技改项目合同能源管理，对耗能重点企业实施能效对标；继续依法淘汰落后生产能力，重点抓好水泥企业落后产能关停淘汰工作。积极发展煤层气等清洁能源，加大和顺、寿阳两大煤层气区块开发力度，扩大过境天然气利用，推进东山各县与灵石城市天然气管网建设，加快市城区人工煤气、管道液化气置换天然气工作，确保完成3万余户人工煤气和1万余户液化气用户置换，消除安全隐患。加快汽车加气站建设，加强燃气使用泄漏报警、巡检等安全基础建设。公共机构要增强节能意识，实现年度人均综合能耗目标。

　　强化减排治污。严格污染减排治理，实施污染减排、污染防治“双百”工程。狠抓电力、水泥行业脱硝项目建设，确保重点排污企业脱硝设施按期投运。加大城区燃煤锅炉治理力度，扩大集中供热覆盖面，全市集中供热普及率提高1个百分点，达到85%；推进城市污水处理提标升级、管网配套和中水回用工程建设，污水处理率达到93%；加快城镇垃圾处理厂建设，垃圾处理率提高9.5个百分点，达到54%。抓好农村环境连片整治，开展规模畜禽养殖场（小区）污染治理。完成市城区省级环保模范城市创建工作。

　　搞好环境整治。加强城乡环境卫生管理，继续引深“四城联创”，创新环卫工作机制，严格落实机关、企事业单位、沿街店铺的环卫责任，试点推行社区公岗环卫清洁。在1850个重点村实施乡村清洁工程，开展创建清洁示范系列活动。市城区、介休市申报创建国家卫生城市，灵石县确保获得国家卫生城市命名，左权县力争通过国家卫生县城暗访和综合评审。

　　推进生态建设。以创建国家级园林城市和省级林业生态市为抓手，完成新造林合格面积50万亩以上，林木绿化率增加1.5个百分点以上。推进晋商公园三期、潇河公园二期、社火公园等重点园林绿化工程，完成高速公路和国省道绿化200公里，加快推进汾河介休段综合整治工程，新建和完善6处城郊森林公园和湿地森林公园，太谷、榆次力争通过省级林业生态县验收，左权、灵石启动创建国家园林县城工作，太谷启动创建省级园林县城工作，平遥、祁县通过省级园林县城初审，寿阳、和顺完成创建省级园林县城。开发利用山庄窝铺，扶持发展生态庄园。

　　（六）持之以恒狠抓安全生产，努力促进安全形势稳定好转。

　　坚持发展是第一要务、安全生产也是第一要务，抓发展是政绩、抓安全生产也是政绩的理念，始终保持如临深渊、如履薄冰的态度，敬畏生命、敬畏责任、敬畏制度，全面贯彻安全第一、预防为主、综合治理的方针，进一步强化政府安全监管主体责任，严格落实企业安全生产主体责任，构建严密的责任互动机制。坚持每月例会、市县行政主官挂牌等基本制度，全面加强安全生产风险管理，启动万家企业安全管理专家会诊行动，推动生产经营单位重点查找安全监管基本体制、基本机制、基本责任、基本制度等方面不适应安全生产规律的薄弱环节和突出问题，逐项整改落实，确保安全制度能覆盖最细微环节，从制度和责任落实两个方面根除安全死角盲点。不断加大安全投入，推进科技兴安、安全标准化建设和企业现场管理，推动以“制度落实、责任落实”为重点的新一轮知责、履责、问责教育培训。以道路交通和煤矿安全为重点，狠抓各行业各领域隐患排查专项整治，扎实推进“打非治违”行动，严厉打击私挖滥采行为。坚持食品药品安全全程防控和责任监督，落实安全生产目标责任考核“一票否决制”，严肃查处各类生产安全事故，依法追究责任，坚决遏制重特大事故，努力减少一般事故，杜绝瞒报迟报，促进全市安全生产形势稳定好转。

　　（七）坚定不移推进改革创新，努力增强发展动力活力。

　　坚持把改革创新贯穿政府工作全过程，抓住重点领域关键环节，先行先试、大胆实践，以改革求突破。勇敢推进综改试验。加快科技创新和产业转型两个园区建设，精心实施标杆项目，优先支持晋中经济技术开发区和榆次工业园先行先试。加快“108综合发展廊带”建设，重点实施397个项目。加快城乡建设用地增减挂钩试点、矿业存量土地整合和露天采矿用地改革，盘活农村闲置宅基地等存量土地资源，努力保障建设用地供应，促进土地节约集约利用。积极推动排污权交易、排污权抵押贷款。创新人才引进和培养机制，加快引进和培养高层次创新人才、经济转型紧缺人才、重要学科领军人才，努力建设适应转型跨越发展的人才队伍。

　　不断推动金融创新。用足用好我市被省政府确定为全省规范民间融资试点和全省资本市场改革创新示范区的机遇，探索建立民间融资登记服务中心，健全融资监测体系；积极引进股份制银行、地方法人机构入驻晋中设立分支机构，培育发展注册资本金规模较大、专业性较强的小额贷款公司；设立注册资本金在2亿元以上的融资担保公司，组建注册资本金在10亿元以上的融资再担保公司；继续鼓励设立村镇银行，延伸服务机构，推动符合条件的县级农村信用联社改制农商行；引导民间资金通过股权、债权等投资方式投向实体经济、科技创新和重点工程项目；培育股权交易中介机构，鼓励发展股权投资企业及其管理企业。

　　继续深化各项改革。深入贯彻落实促进中小企业发展政策措施，不折不扣地落实支持中小企业发展资金，全面落实结构性减税和各项服务优惠政策。积极推进优质企业在场外交易市场挂牌，筹建天津股权交易所山西区域分市场，加快和顺正邦、凯嘉集团的上市工作。努力服务企业家队伍发展需要，推动民营经济快速健康发展。完善国有资产监管体制，做好晋华、锦纶、轻机等企业破产后续工作，化解格芙兰改制遗留问题，全面铺开厂办大集体企业改制工作，稳步推进药材系统和市直部门国有企业改革。积极推进财税体制改革，统筹推进农村综合改革、集体林权制度改革和事业单位分类改革等改革工作。

　　（八）真情实意改善民生民利，努力提升人民幸福指数。

　　坚持把保障和改善民生作为政府一切工作的出发点和落脚点，放在更加突出的位置，努力让人民群众过上更加幸福美好的生活。扎实做好就业和社会保障工作。加强省级创业型城市和充分就业社区建设，重点保障高校毕业生和农村劳动力转移创业就业，动态消除零就业家庭，转移农村劳动力3.87万人，解决就业困难人员就业2459人。统筹推进城乡社会保障体系建设，引导更多非公有制经济组织从业人员、灵活就业人员和农民工参加社会保险，城镇职工基本养老、医疗、失业、工伤、生育保险参保率达到95%以上；企业退休人员基本养老金提高10%，城乡居民基础养老金提高10元、达到65元；城镇居民医保和新农合财政补助标准由每人每年240元提高到280元，逐步实现新农合持卡就诊、补偿“一卡通”；城镇职工和居民基本医疗保险最高支付限额分别提高到48万元、26万元，参保人员门诊慢性病种由13种增加到17种；城乡低保保障标准每人每月分别提高30元、24元，全市平均标准达到348元、172元。认真落实收入分配制度改革政策，按规定提高最低工资标准。新开工建设保障房1.72万套，基本建成19710套，改造农村困难家庭危房4811户，加强保障房阳光租售、运营、退出等管理，确保困难群众有房住、住房质量有保障。继续加强住房公积金扩面征缴，扎实做好项目贷款试点工作，确保公积金安全有效使用。

　　全面推进社会事业发展。努力办好人民满意的教育，推进义务教育均衡发展、教育信息化以及中小学德育三项国家级改革试点工作，统筹各级各类教育协调发展，持续改造农村薄弱学校，启动市城区中小学新改扩建工程，市政府、开发区、榆次区共同筹集资金，力争用三年时间形成布局合理、规范发展的新格局；建设18所省级和53所市级公办标准化幼儿园，改扩建50所村级幼儿园；加快发展现代职业教育，加强中高职教育的有效衔接；创建35所数字化校园示范校；培养培训500名学科带头人，实施3.5万名教师教学能力大练兵，公开招聘一批公办幼儿教师。深化医药卫生体制改革，加强乡村医生队伍建设，巩固完善基本药物制度和基层医疗卫生机构运行新机制，新增3个公立医院改革试点县，促进城乡居民享受基本公共卫生服务均等化。继续打造城市“500米健身圈”，为70个社区建设100个健身场地。进一步做好人口和计划生育工作，稳定低生育水平，提高人口素质。大力发展福利和慈善事业，为5000名残疾人实施康复救助，加快发展民办养老服务机构，积极开展居家养老、社区养老服务。落实各级政府的文物保护责任，支持国防和军队建设，发展妇女儿童、老龄、档案和红十字会等事业，做好双拥、优抚安置、人防、民族、宗教、外事、侨务、对台、气象、史志、科普、地震等工作。

　　深入推进“平安晋中”建设。强化网格化基层社会服务管理，加快43个社会管理创新项目建设，重点推进各类社会服务管理功能网格融合共建，规范“五级”网格平台运行管理，强化社区村组功能，建立横向到边、纵向到底、纵横交错、有机联系的网格化体系。扎实推进“六网覆盖”工程，构建立体化社会治安防控体系，依法打击各种违法犯罪行为，始终保持对严重刑事犯罪活动的严打高压态势，保障人民群众生命财产安全。严格落实信访责任制，完善矛盾纠纷排查调解机制，畅通和规范群众诉求表达、利益协调、权益保障渠道，促进社会公平正义，维护社会和谐稳定。加强公路治超工作。强化防灾减灾能力和应急管理，妥善应对各类突发事件。广泛开展群众性精神文明创建活动，积极推进移风易俗，大力加强社会公德、职业道德、家庭美德和个人品德教育，弘扬和践行“明礼诚信、开放包容、艰苦奋斗、唯实唯先”的晋中精神，形成良好的社会风尚。

　　各位代表！

　　面对新的形势和任务，政府一定要更加重视自身建设，牢记宗旨，勤勉尽职，努力建设人民满意的创新型、廉洁型、法治型、服务型政府。创新政府服务。推进新一轮政府机构改革，进一步简政放权，转变职能，推进政企、政资、政事、政社分开，真正把政府不该管、管不了也管不好的事情交给企业、市场和社会组织，努力做到不“缺位”、“错位”、“越位”。进一步优化政务环境，深化行政审批“两集中、两到位”改革，提升联合审批服务水平，严厉查处一切损害发展环境的违法违纪行为。严格实行目标责任制考核，提高执行力和公信力。努力改进文风会风，精简会议文件，大兴调查研究，深入一线，体察民情，丰富工作思路，完善发展措施，真正学会做群众工作，切实解决人民群众的实际困难和根本问题。坚持依法行政。加强法治政府建设，严格依照法定权限和程序行使权力，坚持科学决策、民主决策、依法决策。强化行政运行监督，自觉接受人民代表大会及其常委会的法律监督、工作监督和人民政协的民主监督，自觉接受社会公众监督和舆论监督，加强行政监察和审计监督，健全权力运行制约和监督体系。加强信息化建设，深化政务公开，推进财政预决算公开和“三公”经费公开，完善政府信息发布制度，让权力在阳光下运行。加强廉政建设。坚持按制度管权用权，认真履行“一岗双责”廉政建设责任制，严格遵守廉洁自律各项规定，深入开展反腐败斗争，强化重点领域监管，严厉查处违纪违法案件，坚决纠正不正之风，做到干部清正、政府清廉、政治清明。大力弘扬艰苦奋斗优良传统，严格公共财政支出预算，严格执行办公用房建设规定，严格控制财政供养人员，严格“三公”经费支出，继续实行零增长，真正把有限的公共财力用在推动发展、改善民生上。

　　各位代表!

　　时代赋予重任，奋斗铸就辉煌。让我们在省委、省政府和市委的坚强领导下，紧紧依靠和团结带领全市人民，凝心聚力，攻坚克难，开拓创新，争先进位，为加快建设全省“四化”率先发展区而不懈努力！
